# Tonic Endocannabinoid Signaling Gates Synaptic Plasticity in Dorsal Raphe Nucleus Serotonin Neurons Through Peroxisome Proliferator-Activated Receptors

**DOI:** 10.3389/fphar.2021.691219

**Published:** 2021-06-28

**Authors:** Saida Oubraim, Ruixiang Wang, Kathryn A Hausknecht, Roh-Yu Shen, Samir Haj-Dahmane

**Affiliations:** ^1^ Department of Pharmacology and Toxicology, Jacobs School of Medicine and Biomedical Sciences, University at Buffalo, Buffalo, NY, United States; ^2^ Neuroscience Program, University at Buffalo, Buffalo, NY, United States

**Keywords:** endocannabinoid, 2-AG, dorsal raphe, tLTP, PPARγ

## Abstract

Endocannabinoids (eCBs), which include 2-arachidonoylglycerol (2-AG) and anandamide (AEA) are lipid signaling molecules involved in the regulation of an array of behavioral and physiological functions. Released by postsynaptic neurons, eCBs mediate both phasic and tonic signaling at central synapses. While the roles of phasic eCB signaling in modulating synaptic functions and plasticity are well characterized, very little is known regarding the physiological roles and mechanisms regulating tonic eCB signaling at central synapses. In this study, we show that both 2-AG and AEA are constitutively released in the dorsal raphe nucleus (DRN), where they exert tonic control of glutamatergic synaptic transmission onto serotonin (5-HT) neurons. The magnitude of this tonic eCB signaling is tightly regulated by the overall activity of neuronal network. Thus, short term *in vitro* neuronal silencing or blockade of excitatory synaptic transmission abolishes tonic eCB signaling in the DRn. Importantly, in addition to controlling basal synaptic transmission, this study reveals that tonic 2-AG, but not AEA signaling, modulates synaptic plasticity. Indeed, short-term increase in tonic 2-AG signaling impairs spike-timing dependent potentiation (tLTP) of glutamate synapses. This tonic 2-AG-mediated homeostatic control of DRN glutamate synapses is not signaled by canonical cannabinoid receptors, but by intracellular peroxisome proliferator-activated receptor gamma (PPARγ). Further examination reveals that 2-AG mediated activation of PPARγ blocks tLTP by inhibiting nitric oxide (NO), soluble guanylate cyclase, and protein kinase G (NO/sGC/PKG) signaling pathway. Collectively, these results unravel novel mechanisms by which tonic 2-AG signaling integrates network activities and controls the synaptic plasticity in the brain.

## Introduction

In the brain, neurons dynamically regulate the strength of their synaptic inputs by adjusting the parameters of synaptic transmission using various signaling molecules, including endocannabinoids (eCBs), a family of neuroactive lipids (Castillo et al., 2012). The two-best characterized eCB species are 2-arachidonoylglycerol (2-AG) and N-arachidonoylethanolamine (AEA or anandamide) (Devane et al., 1992; Mechoulam et al., 1995; Sugiura et al., 1995). Typically, eCBs are synthesized and released “on demand” from postsynaptic neurons in response to phasic neuronal activation (Kreitzer and Regehr., 2001; Ohno-Shosaku et al., 2001; Wilson and Nicoll, 2001), and/or stimulation of Gq/11-coupled neurotransmitter receptors (Haj-Dahmane and Shen, 2005; Hashimotodani et al., 2005; Maejima et al., 2005). This phasic eCB signaling mediates retrograde modulation of synaptic transmission and plasticity throughout the central nervous system *via* presynaptic cannabinoid 1 receptors (CB1Rs) (Castillo et al., 2012; Ohno-Shosaku and Kano, 2014). In addition to retrograde signaling, phasic eCB release also exerts autocrine control of the intrinsic excitability of both pre- and postsynaptic neurons by regulating several membrane ion channels (Bacci et al., 2004; Gantz and Bean, 2017). Through retrograde and autocrine signaling, phasic eCB release regulates neuronal excitability and gates various forms of synaptic plasticity in the brain (Araque et al., 2017).

In addition to phasic eCB signaling, ample evidence indicates that both 2-AG and AEA are constitutively synthesized and released at central synapses. The presence of tonic 2-AG synthesis is supported by the findings that in the absence of neuronal activation, pharmacological inhibition or genetic deletion of monoacylglycerol lipase (MAGL), the main enzyme that hydrolyzes 2-AG, increases 2-AG levels in the brain (Hashimotodani et al., 2007; Tanimura et al., 2010). Similarly, inhibition of fatty acid amide hydrolase (FAAH), the enzyme that degrades AEA (Deutsch and Chin, 1993), increases brain AEA levels (Cravatt et al., 2001; Booker et al., 2012; Caprioli et al., 2012), thereby indicating tonic AEA synthesis and release. Furthermore, blockade of CB1Rs and inhibition of eCB degradation potentiates and depresses basal synaptic transmission, respectively, indicating that activation of presynaptic CB1Rs exerts a tonic control of basal synaptic transmission (Haj-Dahmane et al., 2018; Hentges et al., 2005; Neu et al., 2007; Roberto et al., 2010). Importantly, numerous preclinical studies have reported alterations of tonic eCB signaling in rodent models of neurological/psychological disorders, such as autism (Földy et al., 2013), Huntington’s disease (Dvorzhak et al., 2013) and epilepsy (Chen et al., 2003). Collectively, these studies strongly suggest that tonic eCB signaling plays an important role in the regulation of normal brain functions and in the pathophysiology of various neurological/psychological disorders.

Although the presence of tonic eCB signaling at central synapses is well documented, its precise physiological roles and the mechanisms by which tonic eCB signaling controls synaptic functions are not well defined. In the present study, we show that, in addition of controlling basal synaptic transmission, tonic eCB signaling serves as a homeostatic mechanism that gates spike-timing dependent plasticity (tLTP) of glutamate synapses in the dorsal raphe nucleus (DRN). This physiological role is not signaled by canonical cannabinoid receptors, but through activation of intracellular peroxisome proliferator-activated receptor gamma (PPARγ) leading to inhibition of nitric oxide (NO) signaling cascades. As such, these results uncover an important and novel mechanism by which tonic eCB signaling controls synaptic function in the brain.

## Materials and Methods

**Brain slices preparation:** The experimental procedures used in the present study were approved by the University at Buffalo Animal Care and Use Committee (IACUC) in accordance with the National Institutes of Health (NIH) Guidelines for the Care and Use of Laboratory Animals. Brain slices containing the DRn were prepared from 6– 8-week-old male Sprague-Dawley rats (Envigo, Indianapolis, IN, United States) using a standard procedure (Wang et al., 2012). Rats were killed by decapitation under isoflurane anesthesia and the brainstem area containing the DRN was isolated. Coronal slices from the DRn (300–350 µm) were cut using a vibratome (Leica VT1200S; Leica Biosystems, St Louis, MO, United States) in ice-cold modified ACSF of the following composition (in mM): 110 choline-Cl, 2.5 KCl, 0.5 CaCl_2_, 7 MgSO_4_, 1.25 NaH_2_PO_4_, 26.2 NaHCO_3_, 11.6 sodium l-ascorbate, 3.1 sodium pyruvate, and 25 glucose, equilibrated with 95% O_2_5% CO_2_. Slices were incubated for 45 min at 35°C and then at room temperature for at least 1 h in a holding chamber containing regular ACSF (in mM): 119 NaCl, 2.5 CaCl_2_, 1.3 MgSO_4_, 1 NaH_2_PO_4_, 26.2 NaHCO_3_, and 11 glucose and continuously bubbled with a mixture of 95% O_2_5% CO_2_. Following recovery, slices were transferred to a recording chamber (Warner Instruments, Hamden, CT, United States) mounted on a fixed upright microscope and continuously perfused (2–3 ml/min) with ACSF saturated with 95% O_2_5% CO_2_ and heated to 30 ± 1°C using a solution heater (Warner Instruments, Hamden, CT, United States).

To examine the role of baseline neuronal network activity in controlling tonic eCB signaling, slices were incubated for 3–4 h in a holding ACSF containing tetrodotoxin (TTX, 1 µM) or Kynurenic acid (KA, 1 mM) to block action potentials and excitatory synaptic transmission, respectively. The electrophysiological recordings were performed after extensive (>1 h) washout of TTX or KA that restores action potential firing or synaptic transmission. For the experiments assessing the impacts of increased tonic 2-AG and AEA signaling on synaptic plasticity, slices were pretreated for 30–45 min with MAGL and FAAH inhibitors JZL184 and PF750, respectively. The electrophysiological experiments were performed 3–4 h after washout of the inhibitors.

**Electrophysiological recordings:** Neurons of the DRn were visualized using a BX 51 Olympus microscope (Olympus Co, Tokyo, Japan) equipped with a 40x water-immersion lens, differential interference contrast and infrared optical filter. Somatic whole-cell recordings were obtained from putative DRN 5-HT neurons with patch electrodes (3–5 MΩ) filled with a solution containing (in mM): 120 potassium gluconate; 10 KCl, 10 Na_2_-phosphocreatine, 10 HEPES, 1 MgCl_2_, 1 EGTA, 2 Na_2_-ATP, and 0.25 Na-GTP; pH 7.3; osmolality 280–290 mOsm/kg. DRN 5-HT neurons were identified by the large after-hyperpolarizing potentials (AHPs), slow evoked firing activity and by the 5-HT_1A_ receptor-induced potassium current/membrane hyperpolarization (Haj-Dahmane, 2001). Using intracellular post-hoc biocytin labeling and tryptophan hydroxylase type 2 (TPH2) immunohistochemistry, we have previously shown that all neurons exhibiting these electrical properties were TPH2 positive (Geddes et al., 2016). Therefore, only neurons that exhibited these features were included in these studies.

All recordings were performed from 5-HT neurons located in the dorsomedial subdivision of the DRN (dmDRN). Excitatory post-synaptic currents (EPSCs) were evoked with single square-pulses (duration = 100–200 µs) delivered at 0.1 Hz with patch pipettes (2–3 mΩ) filled with ACSF and placed (50–100 µm) dorsolateral to the recording sites. In some experiments, to assess the change in PPR, pairs of EPSCs were evoked with an inter-stimulus interval of 30 ms. The intensity of the stimulus was adjusted to evoke 75% of the maximal amplitude of EPSCs. α-amino-3-hydroxy-5-methyl–4-isoxazolepropionic acid (AMPA) receptor-mediated EPSCs were recorded from neurons voltage-clamped at -70 mV in the presence of GABAA receptor antagonist picrotoxin (100 µM) and glycine receptor antagonist strychnine (20 µM). Membrane currents and voltages were amplified with an Axoclamp 2B or Multiclamp 700B amplifier (Molecular Devices, Union City, CA, United States). Membrane currents were filtered at 3 kHz, digitized at 20 kHz with Digidata 1,440 (Molecular Devices), and acquired using the pClamp 10.7 software (Molecular Devices). The cell input resistance and access resistance (10–20 mΩ) were monitored throughout the experiment using 5 mV hyperpolarizing voltage steps (500 ms duration) and the recordings were discarded if the input and series resistance change by more than 20–30%.

To examine whether glutamatergic synapses onto DRN 5-HT neurons exhibit activity-dependent synaptic plasticity, we used a stimulation pattern that reliably induces tLTP (Haj-Dahmane et al., 2017). This protocol consists of pairing trains of five bursts of presynaptic stimulation paired with bAPs delivered at 5 Hz. Each burst is composed of three presynaptic stimuli (50 Hz) paired with three backpropagating action potentials (bAPs) (50 Hz) with a delay of 5–10 ms. Action potentials were evoked by injection of depolarizing somatic currents (1.5–2 nA, 2 ms duration) in the current clamp mode. After obtaining a stable recording of AMPAR-EPSCs for at least 10 min, the recordings were switched to the current clamp mode and 20 trains of five bursts were delivered at 0.1 Hz. Immediately after the administration of the stimulation protocol, the recordings were switched back to the voltage clamp mode.

**Data analysis:** eEPSCs were analyzed using Clampfit 10.2 software (Molecular Devices). The amplitude of eEPSCs was determined by measuring the average current during a 2 ms time window at the peak of each eEPSC and subtracted from the baseline current determined during a 5 ms time window before the stimulus artifact. All eEPSC amplitudes were normalized to the mean baseline amplitude recorded for at least 10 min before administration of a drug or the tLTP pairing protocol. In paired pulse experiments the paired pulse ratio (PPR = EPSC2/EPSC1) were averaged for at least 60 consecutive trials before and 30–40 min after administration of the tLTP protocol. To determine the coefficient of variation (CV), the standard deviation (SD) and the mean amplitude of eEPSCs were calculated for at least 60 consecutive trials before and during the tLTP. The CV was then given by the following ratio (SD)/(EPSC mean amplitude). Statistical analysis was performed using Origin 8.0 software (OriginLab Co, Northampton, MA, United States). The results in the text and figures are expressed as mean ± SEM. Statistical comparisons were conducted using the Student’s paired *t*-test and for within group comparison and independent *t*-test for comparison between group. Statistical significance was set at *p* < 0.05.

**Chemicals:** Chemicals and salts were obtained from Fisher Scientific (Pittsburgh, PA, United States). N-(Piperidin-1-yl)-5-(4-iodophenyl)-1-(2,4-dichlorophenyl)-4-methyl-1H-pyrazole-3-carboxamide (AM 251), 4-[Bis(1,3-benzodioxol-5-yl) hydroxymethyl]-1-piperidinecarboxylic acid 4-nitrophenyl ester (JZL184), N-Phenyl-4-(3-quinolinylmethyl)-1-piperidinecarboxamide (PF750), Picrotoxin and strychnine were purchased from Tocris Bioscience (Minneapolis, MN, United States). 5-{[4-((3,4-Dihydro-6-hydroxy-2,5,7,8-tetramethyl-2H-1-benzopyran-2-yl) methoxy) phenyl] methyl}-2,4-thiazolidinedione (Troglitazone), 2-Chloro-5-nitro-N-phenylbenzamide (GW9662), and (S)-Nitroso-N-acetylpenicillamine (SNAP) were purchased from Sigma-Aldrich (St Louis, MO, United States).

## Results

### Tonic eCB Signaling Controls Basal Glutamatergic Transmission in the DRN

**FIGURE 1:**
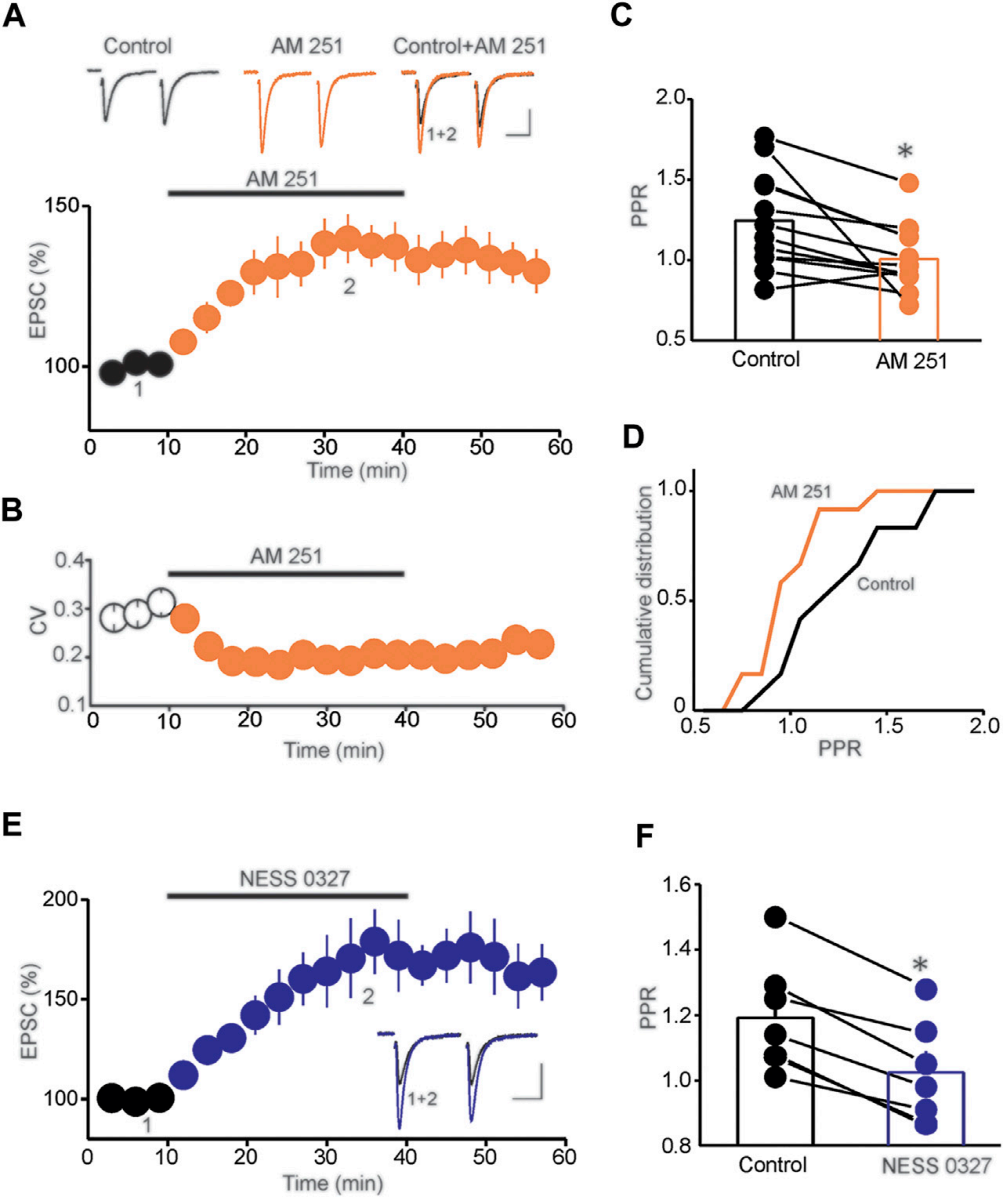
Tonic eCB signaling controls baseline glutamatergic synaptic transmission. **(A)** Blockade of CB1Rs with AM 251 potentiates the amplitude of eEPSCs. Lower panel is a summary graph of the potentiation of eEPSCs induced by AM 251 (3 µM; *n* = 15). Upper panel depicts sample eEPSC traces taken before and during AM 251 application. Calibration bars: 50 pA, 20 ms. **(B)** Summary graph of the effect of AM 251 on the CV (*n* = 15). **(C)** Summary histogram of the average PPR determined in control and during AM 251 administration (*p < 0.05; paired *t*-test; *n* = 12). **(D)** Cumulative distribution of the PPR of EPSCs determined in the control condition and in the presence of AM 251 (*p* < 0.01; K-S test; *n* = 16). **(E)** The neutral CB1R antagonist NESS 0327 (1 µM; *n* = 12) potentiates the amplitude of eEPSCs. Inset illustrates superimposed eEPSC traces taken before and during NESS application. Calibration bars: 50 pA, 20 ms. **(F)** Summary histogram of the effect of NESS 0327 on PPR (*p < 0.05; paired *t*-test; *n* = 7).

To examine the role of tonic eCB signaling in controlling glutamatergic synaptic transmission onto DRN 5-HT neurons, we performed *ex-vivo* whole-cell patch-clamp recordings from putative DRN 5-HT neurons and assessed the impact of the CB1R antagonist/inverse agonist AM 251 on the baseline amplitude of evoked excitatory postsynaptic currents (eEPSCs). Consistent with a previous report (Haj-Dahmane et al., 2018), we found that application of AM 251 (3 µM) significantly increased the amplitude of eEPSCs (140.9 ± 6.4% of baseline, *n* = 15, *p* < 0.01, paired *t*-test, AM251 vs. baseline, Figure 1A). The increase of eEPSC amplitude was accompanied with a significant decrease in coefficient of variation (CV) (CV control = 0.27 ± 0.016, CV AM 251 = 0.15 ± 0.02, *n* = 15, *p* < 0.02, paired *t*-test, control vs. AM 251, Figure 1B) and paired-pulse ratio (PPR) of eEPSCs (PPR control = 1.31 ± 0.06, PPR AM 251 = 1.02 ± 0.05, *n* = 12, *p* < 0.05 paired *t*-test, control vs. AM 251, Figure 1C), which resulted in a leftward shift of the cumulative distribution of PPR (n = 16, Kolmogorov-Smirnov test, *p* < 0.01, Figure 1D). Collectively, these results demonstrate that AM 251 potentiates the amplitude of eEPSCs by increasing glutamate release in the DRn.

In addition to being a mixed antagonist/inverse agonist of CB1Rs, AM 251 is also an agonist of non-cannabinoid receptors, including the orphan receptor GPR 55 (Pertwee, 2007; Kapur et al., 2009). Therefore, to further assess whether the potentiation of eEPSCs is mediated by blockade of CB1Rs, we tested the effects of a neutral and selective CB1R antagonist NESS 0327 on the amplitude of eEPSCs. Similar to the effect of AM 251, bath application of NESS 0327 (1 µM) increased eEPSC amplitude (173.32 ± 9.7% of baseline, *n* = 12, *p* < 0.01 paired *t*-test, NESS 0327 vs. baseline, Figure 1E) and significantly reduced the PPR of eEPSCs (PPR control = 1.21 ± 0.02, PPR NESS 0327 = 1.01 ± 0.05, *n* = 7, *p* < 0.05 paired *t*-test, control vs. NESS 0327, Figure 1F), thereby demonstrating that blockade of CB1Rs potentiates glutamatergic synaptic transmission onto DRn 5-HT neurons. Such results also indicate tonic activation of CB1Rs that depresses the probability of glutamate release in the DRn.

**FIGURE 2:**
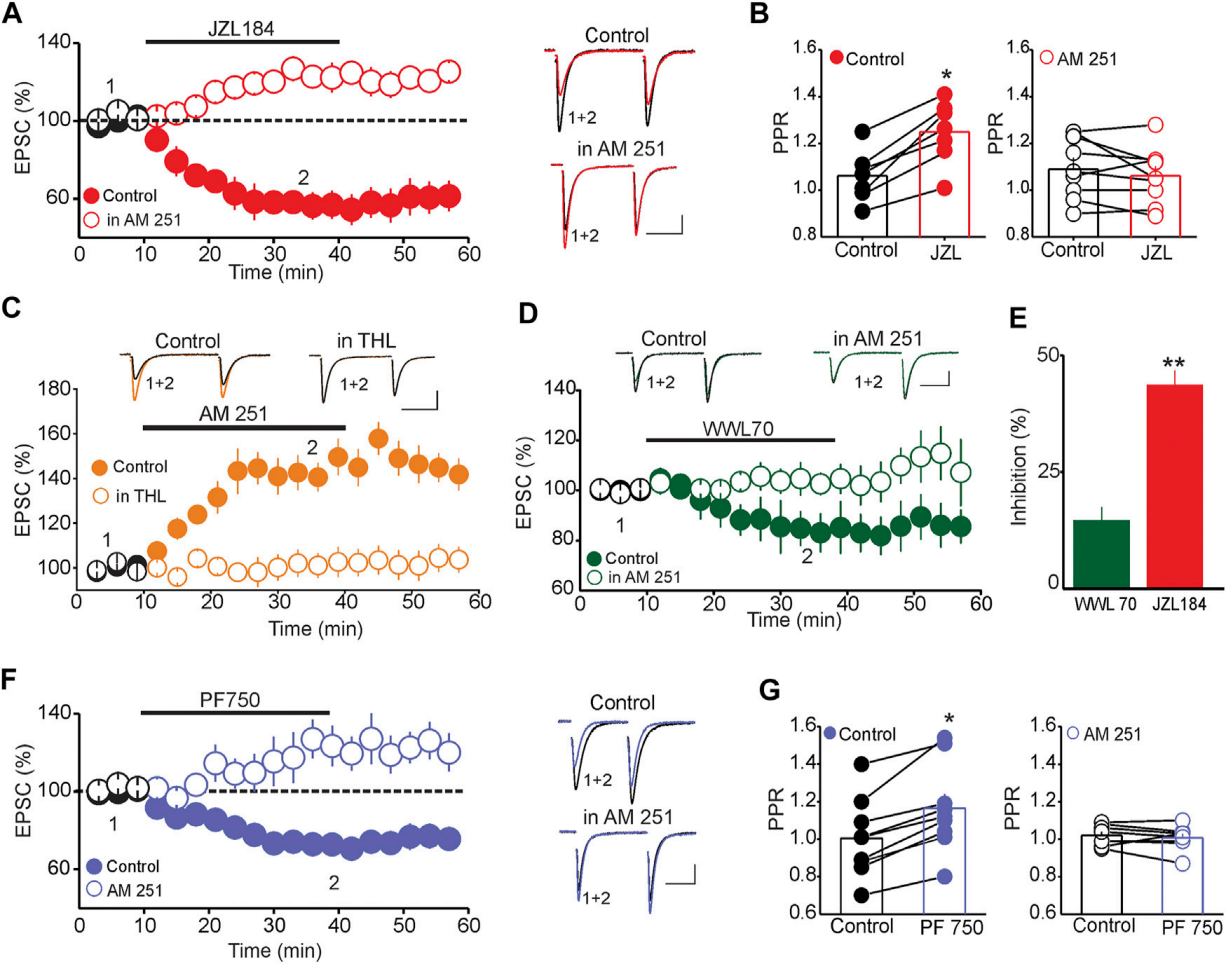
Tonic 2-AG and AEA signaling modulate the strength of DRn glutamate synapses. **(A)** Inhibition of 2-AG degradation depresses the eEPSC amplitude *via* a CB1R-depedent mechanism. Left panel is a summary graph of the effect of JZL184 (1 µM) on the amplitude of eEPSCs in the control condition (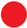; *n* = 12) and in the presence of 3 µM AM 251 (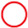; *n* = 10). Right graph illustrates superimposed eEPSC traces taken at the time indicated by numbers in the left panel. Calibration bars: 25 pA, 20 ms. **(B)** Inhibition of 2-AG metabolism increases PPR of eEPSCs *via* CB1Rs. Left graph is summary histogram of PPR of eEPSCs obtained in control slices before (control) and during JZL (1 µM) administration (*p < 0.05; paired *t*-test; *n* = 9). Right panel is summary histogram of PPR of eEPSCs obtained in slices treated with AM 251 (3 µM) before (control) and during JZL administration. **(C)** Inhibition of 2-AG synthesis abolishes the AM 251-induced potentiation of EPSCs. Lower panel is a summary graph of the potentiation of EPSCs amplitude induced by AM 251 (3 µM) in control slices (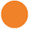; *n* = 9) and in slices treated with THL (10 µM; 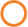; *n* = 9). Upper panel represents superimposed eEPSC traces collected during the time points indicated by numbers in the lower panel. **(D)** Inhibition of ABHD6 depresses the amplitude of eEPSCs through CB1Rs. Lower panel illustrates the magnitude of the depression of EPSCs induced by ABHD6 inhibitor WWL70 (3 µM) in the absence (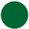; *n* = 8) and presence of AM 251 (3 µM; 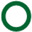; n = 6). Upper graph depicts superimposed EPSC traces collected at the time points indicated by numbers in the lower panel. **(E)** Summary of the average depression of the eEPSC amplitude induced by WWL 70 (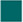) and JZL 184 (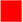; **p < 0.01 JZL184 vs. WWL70; unpaired *t*-test). **(F)** Inhibition of AEA degradation depresses the amplitude of EPSCs through CB1Rs. Left panel is a summary of the effect of the FAAH inhibitor PF750 (3 µM) on eEPSCs obtained in control (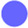; *n* = 10) and in slices treated with AM 251 (3 µM; 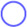; *n* = 8). Right graph illustrates sample eEPSC traces collected at the time indicated by numbers in left panel. **(G)** Inhibition of FAAH increases the PPR of EPSCs. Summary histograms of the average PPR of eEPSCs obtained before and during PF750 application in control slices (left panel; *p < 0.05, paired *t*-test; *n* = 10) and in slices treated with 3 µM AM 251 (right panel, *n* = 8). Calibration bars: 25pA, 20 ms.

Mechanistically, tonic activation of CB1Rs can be mediated by either tonic release of eCBs or constitutively active CB1Rs in the absence of endogenous ligands (Kenakin, 2004; Pertwee, 2005). To distinguish between these possibilities, we examined whether inhibiting eCB degradation, which presumably enhances synaptic eCB levels, could depress the amplitude of eEPSCs. To that end, we tested the impact of inhibiting MAGL on the amplitude of eEPSCs and the probability of glutamate release. Administration of the MAGL inhibitor JZL184 (1 µM) depressed the amplitude of eEPSCs (56.83 ± 7.01% of baseline, *n* = 12, *p* < 0.01, paired t-test, JZL184 vs. baseline, Figure 2A) and increased the PPR of eEPSCs (Baseline = 1.06 ± 0.04, JZL184 = 1.25 ± 0.05, *n* = 8, *p* < 0.02 paired *t*-test, JZL184 vs. baseline, Figure 2B, left panel). Blocking CB1Rs with AM 251 prevented the JZL 184-induced depression of EPSC amplitude (EPSC amplitude = 115.89% of baseline, *n* = 10, n. s vs. baseline, Figure 2A) and increase in PPR (PPR AM 251 = 1.09 ± 0.06, PPR JZL 184 = 1.04 ± 0.04, *n* = 9, n. s. vs. AM 251, Figure 2B, right panel), indicating that these effects were mediated by a CB1R-dependent mechanism. To further confirm that 2-AG is constitutively synthesized and released in the DRn, we also examined the impact of inhibiting diacylglycerol lipase alpha (DAGLα), the main enzyme of 2-AG synthesis on the magnitude of the potentiation of eEPSCs induced by blockade of CB1Rs. Inhibition of DAGLα with tetrahydrolipstatin (THL, 10 µM), which has no effect on baseline excitatory synaptic transmission (Haj-Dahmane and Shen, 2005; Haj-Dahmane et al., 2018) prevented the AM 251-induced potentiation of eEPSCs (Control = 147.8 ± 6.7% of baseline, THL = 102.7 ± 5.6% of baseline, *n* = 9, *p* < 0.01 independent *t*-test, THL vs. control, Figure 2C). Essentially, a similar effect was obtained using a structurally different DAGLα inhibitor RHC-80267 (50 μM) (RHC-80267 = 99.7 ± 5.6% of baseline, *n* = 8, data not shown). Collectively, these results indicate that the constitutive activation of CB1Rs at glutamate synapses impinging onto DRn 5-HT neurons is mainly mediated by tonic 2-AG release.

Because 2-AG can also be metabolized by α/β-hydrolase domain-containing 6 (ABHD6) (Blankman et al., 2007; Muccioli et al., 2007), and inhibition of this metabolic pathway enhances brain 2-AG levels and facilitates 2-AG signaling (Marrs et al., 2010), we tested whether this pathway can control 2-AG metabolism in the DRn. The result showed that pharmacological inhibition of ABHD6 with WWL70 (3 µM) induced a small, albeit significant, inhibition of eEPSC amplitude (85.5 ± 5.49% of baseline, *n* = 8, *p* < 0.05**,** paired *t*-test, WWL70 vs. baseline, Figure 2D), which was readily blocked by the CB1R antagonist AM 251 (101. 5 ± 4.55% of baseline, *n* = 6, n. s. vs. baseline, Figure 2D). The magnitude of the depression of eEPSC amplitude induced by ABHD6 inhibition was significantly smaller than that induced by MAGL inhibitor (WWL 70 = 14.5 ± 4.5% of baseline, JZL = 43.55 ± 4.2% of baseline, *n* = 8, *p* < 0.01 independent *t*-test, JZL184 vs. WWL 70, Figure 2E), suggesting that the ABHD6 pathway plays a minor role in controlling 2-AG hydrolysis in the DRn.

We next tested whether tonic AEA signaling also regulates the strength of glutamate synapses in the DRn by examining the effect of FAAH inhibition on the amplitude of eEPSCs. Administration of the FAAH inhibitor, PF 750 (3 µM), significantly depressed the amplitude of eEPSCs (72.65 ± 7.05% of baseline, *n* = 10, *p* < 0.05, paired *t*-test, PF750 vs. baseline, Figure 2F) and increased the PPR (PPR control = 1.01 ± 0.06, PPR PF 750 = 1.17 ± 0.05, *n* = 10, *p* < 0.05 paired *t*-test, Figure 2G, left panel). These effects were readily blocked by the CB1R antagonist AM 251 (109.61 ± 7.05% of baseline, *n* = 8, n. s. vs. baseline, Figure 2F; PPR AM 251 = 1.04 ± 0.03, PPR PF750 = 1.00 ± 0.02, *n* = 8, n. s, Figure 2G, right panel), thereby indicating that tonic AEA depresses glutamatergic synapses onto DRn 5-HT neurons *via* a CB1R-dependent mechanism.

### Baseline Network Activity Controls Tonic eCB Signaling in the DRN

**FIGURE 3:**
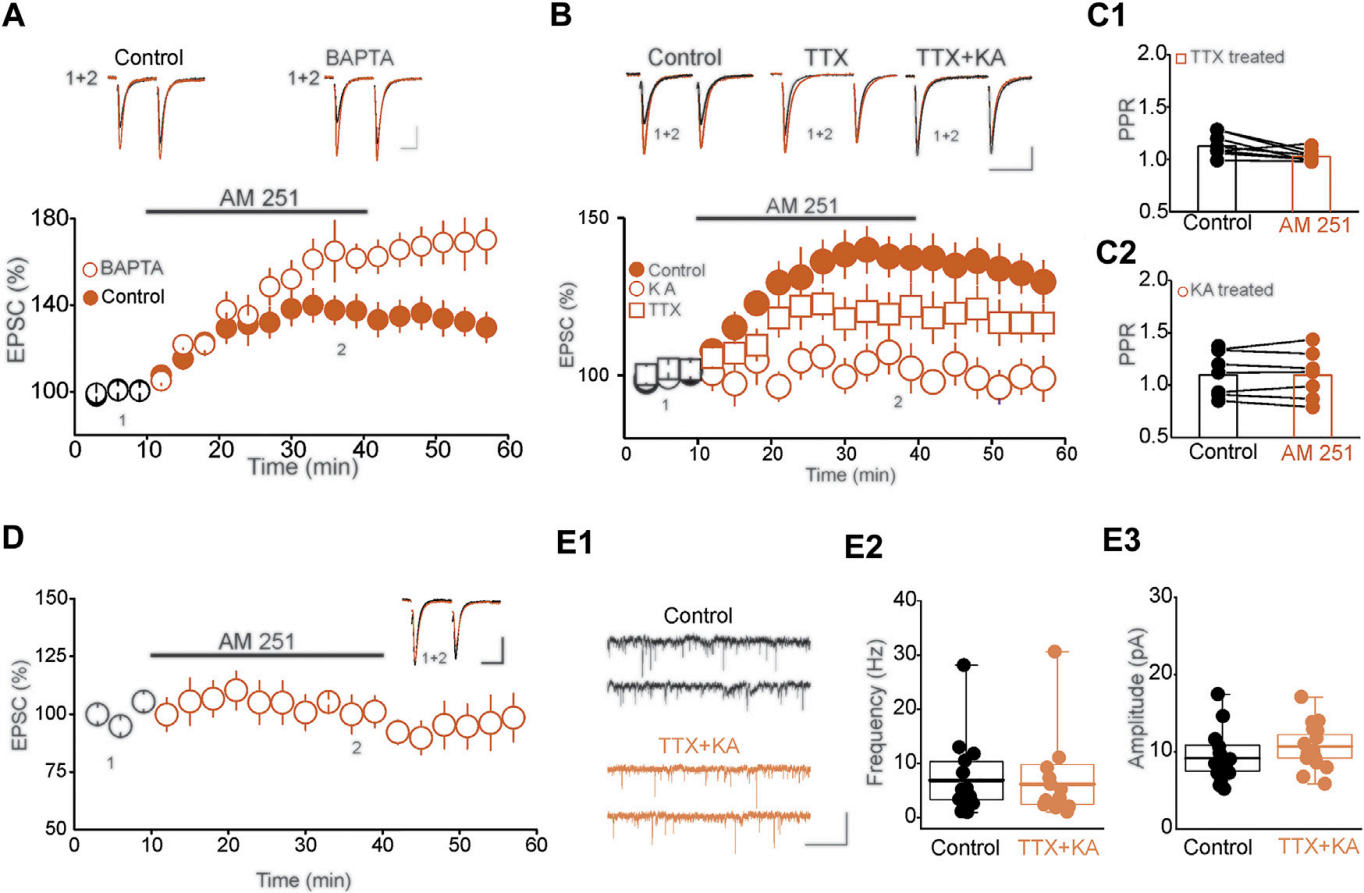
Baseline network activity controls tonic eCB signaling in DRn 5-HT neurons. **(A)** Tonic eCB signaling is not mediated by *de novo* calcium-dependent eCB synthesis/release. Left panel is a summary of the potentiation of eEPSCs induced by AM 251 (3 µM) and recorded with internal solution containing a low concentration (0.1 mM; control; 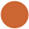; *n* = 9) and a high concentration (10 mM; 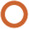; *n* = 9) of BAPTA. Right panel depicts the corresponding eEPSC traces taken at the time points indicated by numbers in the left graph. Calibration bars: 50 pA, 20 ms. **(B)** Baseline network activity controls tonic eCB signaling. Lower panel illustrates the magnitude of the potentiation of the eEPSC amplitude induced by AM 251 (3 µM) in slices incubated in normal ACSF (control; 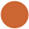; *n* = 10), and in ACSF containing TTX (1 µM; 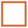; *n* = 9) or KA (1 mM; 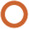; *n* = 10). Upper panel illustrates superimposed eEPSC traces taken at the time points indicated by numbers in the lower graph. Calibration bars: 25 pA, 20 ms. **(C1)** and **(C2)** are summary histograms of the PPR of eEPSCs obtained before (control) and during administration of AM 251 (3 µM) in slices stored in ACSF containing TTX (1 µM) or KA (1 mM), respectively. **(D)** Blockade of synaptic transmission abolishes tonic eCB signaling. Left panel illustrates a summary of the effect of AM 251 (3 µM) on the eEPSC amplitude in DRn slices incubated in ACSF containing 0 mM Ca_2+_ and 6 mM Mg_2+_ (*n* = 8). Inset depicts superimposed EPSC traces taken at the time points indicated in the lower graph. Calibration bars: 50 pA, 25 ms. **(E)** Short-term blockade of neuronal activity has no effects on the frequency and amplitude of mEPSCs **(E1)** Sample current traces of mEPSCs recorded in slice incubated in control condition (upper panel) and in TTX (1 µM) + KA (1 mM). Calibration bars: 20 pA, 500 ms. Summary histograms of the mEPSC frequency **(E2)** and amplitude **(E3)** recorded in control (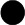; *n* = 15) and TTX + KA (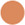; *n* = 15) treated DRn slices.

Synaptic activation and the subsequent increase in postsynaptic intracellular calcium [Ca ^2+^]_i_ triggers eCB synthesis and release, which mediates retrograde inhibition of synaptic transmission (Kreitzer and Regehr, 2001; Ohno-Shosaku et al., 2001). Consequently, it is conceivable that the observed tonic eCB could simply reflects *de novo* phasic eCB synthesis induced by the stimulation protocol used to monitor the effect of CB1R blockade on synaptic transmission. We tested this possibility by examining the impact of chelating [Ca ^2+^]_i_, using patch pipette solution containing high concentration of the fast Ca^2+^ chelator BAPTA on the magnitude of AM-251-induced potentiation of eEPSCs. If tonic eCB signaling were to reflect *de novo* Ca^2+^-dependent eCB synthesis, buffering post-synaptic [Ca ^2+^]_i_ should prevent the AM 251-induced potentiation of eEPSCs. Remarkably, loading postsynaptic DRn neurons with BAPTA (10 mM), a manipulation that prevents fast transient increase in [Ca ^2+^]_i_, as assessed by the blockade of the slow after hyperpolarizing potential (sAHP, data not shown), did not block, but rather slightly enhanced the AM 251-induced potentiation of eEPSCs (0.1 mM BAPTA = 140. 35 ± 7.4% of baseline, 10 mM BAPTA = 157.6 ± 9.3% of baseline, *n* = 9, *p* < 0.05, paired *t*-test, AM251 vs. baseline, Figure 3A). Such results indicate that tonic eCB signaling in the DRn cannot be attributed to *de novo* eCB synthesis and that transient increase in [Ca ^2+^]_i_ is not required for tonic eCB synthesis and release.

To determine the mechanisms that regulate tonic eCB signaling, we examined whether baseline network activity is sufficient to drive constitutive eCB synthesis and release in the DRn. To that end, we first assessed the impact of activity deprivation on tonic activation of CB1Rs. To inhibit network activity, we incubated DRn slices for 4–5 h in ACSF containing the sodium channel blocker tetrodotoxin (TTX, 1 µM). After extensive wash out and action potential recovery, the magnitude of the AM 251-induced potentiation of eEPSCs was assessed in slices preincubated with and without TTX. Blocking neuronal firing significantly reduced the magnitude of AM 251-induced potentiation of eEPSCs (Control = 138.9 ± 6.4% of baseline, TTX = 119.56 ± 7.25% of baseline, *n* = 9, *p* < 0.05, intendent *t*-test, control vs. TTX, Figure 3B) and prevented the associated decrease in PPR (Control = 1.12 ± 0.045, AM 251 = 1.09 ± 0.07, *n* = 8, n. s, Figure 3C1). This finding indicates that action-potential driven neuronal activity contributes, at least in part, to tonic eCB synthesis and release.

The observation that a significant tonic eCB signaling persists after blockade of neuronal activity, raised the possibility that action potential-independent excitatory synaptic drive may also contribute to tonic eCB. To test this possibility, we assessed the impact of blocking excitatory synaptic transmission for 4–5 h using KA, non-selective low affinity ionotropic glutamate receptor antagonist on the magnitude of AM 251-induced potentiation of eEPSC amplitude. Incubating DRn slices in KA (1 mM) abolished the AM 251-induced potentiation of EPSCs (Control = 138.9 ± 6.4% of baseline, KA = 102.4 ± 7.8% of baseline, *n* = 10, n. s. vs. baseline, *p* < 0.01 independent *t*-test, TTX vs. control, Figure 3B) and the associated decrease in PPR (Control = 1.05 ± 0.034, AM 251 = 1.02 ± 0.038, *n* = 6, n. s, Figure 3C2). Next, we examined the effects of blocking synaptic transmission by incubating DRn slices in ACSF containing 0 mM [Ca^2+^] and high [Mg^2+^] (6 mM), on the AM 251-induced potentiation of eEPSC amplitude. We found that short-term (4–5 h) blockade of synaptic transmission abolished the AM251-induced potentiation of eEPSC amplitude (97.72 ± 7.3% of baseline, *n* = 8, n. s. vs. baseline, Figure 3D). Because short-term neuronal silencing and blockade of synaptic transmission could alter both glutamate release and the function of AMPARs (i.e. synaptic scaling), the lack of AM 251 effect on eEPSCs induced by these conditions may be due to synaptic scaling and not inhibition of tonic eCB signaling. Therefore, we tested this possibility by examining the impact of short-term neuronal inactivation on the frequency and amplitude of mEPSCs. We found that pretreatment of DRn slices with TTX (1 µM) and KA (1 mM) did not affect the frequency (Control = 6.85 ± 1.8 Hz, TTX + KA = 6.14 ± 1.9, *n* = 15, *p* > 0.05 independent *t*-test; control vs. TTX + KA, Figure 3E1–E2) nor the amplitude of mEPSCs (Control = 9.20 ± 0.86 pA; TTX + KA = 10.72 ± 0.78, *n* = 15, *p* > 0.05 independent *t*-test, Figure 3E3), indicating that short-term neuronal silencing did not induce homeostatic scaling of glutamate synapses in the DRn. Importantly, such results also indicate that the blockade of AM 251-induced potentiation of EPSCs in response to neuronal silencing could not be attributed to alterations of the basal glutamatergic transmission. Collectively, these results show that action potential-independent miniature excitatory synaptic transmission is sufficient to drive tonic eCB synthesis and release at DRn glutamatergic synapses.

### Tonic 2-AG Signaling Controls Spike-Timing Dependent Long-Term Potentiation in the DRN

**FIGURE 4:**
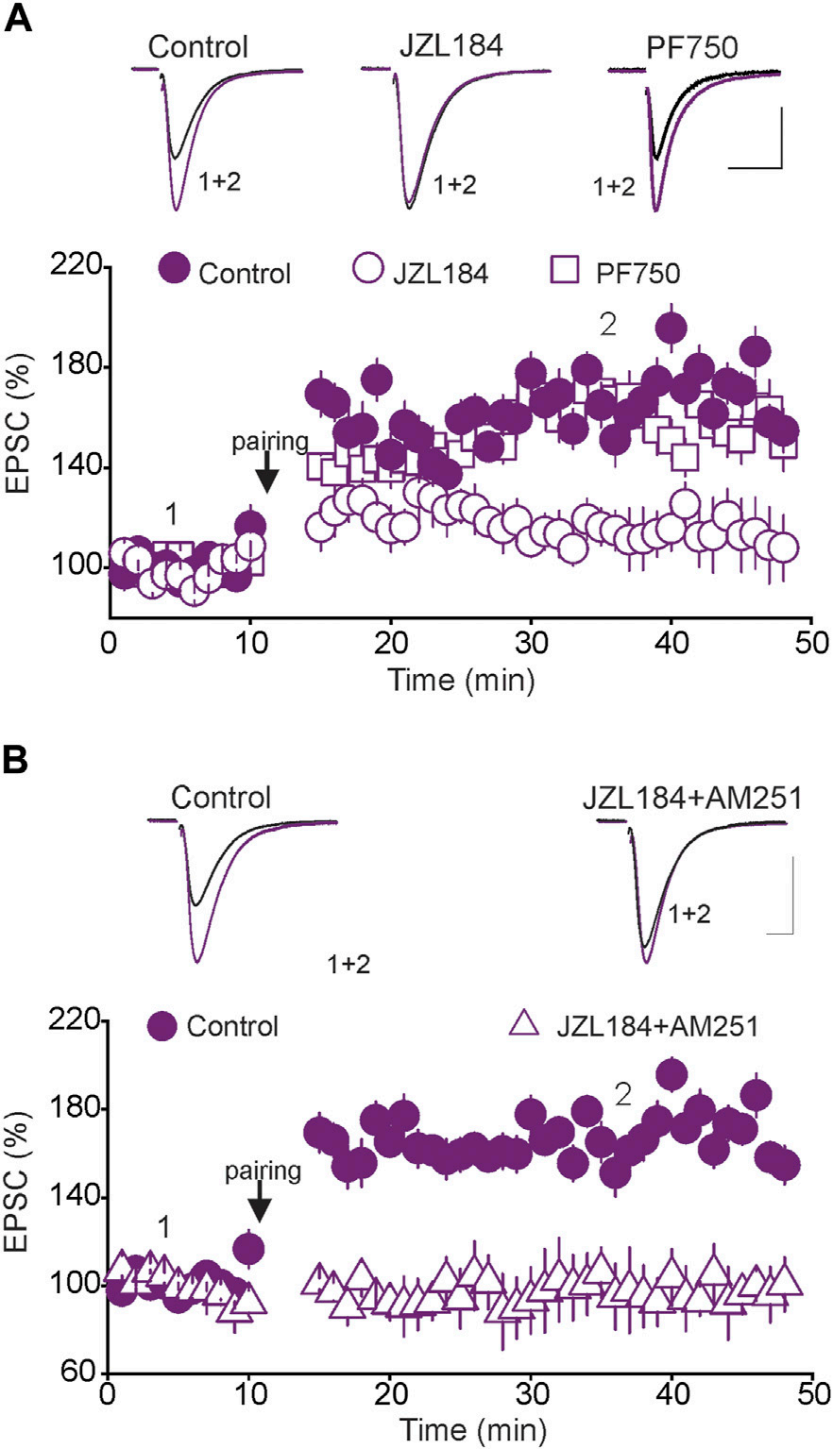
The blockade of tLTP by tonic 2-AG signaling is not mediated by CB1Rs. **(A)** Inhibition of MAGL, but not FAAH abolishes the tLTP. Lower graph is a summary of the tLTP obtained in control DRn slices (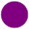; *n* = 9), and in slices incubated with JZL 184 (1 µM; 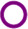; *n* = 9) or PF 750 (3 µM; 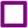; *n* = 9). Upper panel illustrates superimposed eEPSC traces taken before pairing and during tLTP. **(B)** Blockade of CB1Rs does not rescue the tLTP in DRn slices treated with the MAGL inhibitor JZL184 (1 µM). Lower graph is a summary of the tLTP obtained in control slices (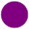; *n* = 9) and in slices treated with JZL184 + AM 251 (3 µM, 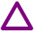; *n* = 9). Upper panel illustrates superimposed eEPSC traces taken before and during the tLTP. Calibration bars: 100 pA, 5 ms.

Although the presence of tonic eCB signaling has been reported at synapses in various brain regions (Hentges et al., 2005; Neu et al., 2007; Haj-Dahmane et al., 2018), its precise physiological roles remain unknown. For instance, it is unknown whether manipulations that transiently alter tonic eCB signaling affect the rules governing synaptic plasticity. We have previously shown that glutamatergic synapses in the DRN undergo activity-dependent synaptic plasticity (Haj-Dahmane et al., 2017). Indeed, pairing presynaptic stimulation with back-propagating action potentials (bAPs) elicits spike-timing-dependent long-term potentiation (tLTP) of glutamatergic synapses in the DRN (Haj-Dahmane et al., 2017). To test whether tonic eCB signaling controls tLTP, we examined the effects of transient inhibition of MAGL and FAAH, manipulations known to increase tonic 2-AG and AEA levels, respectively, on the expression and magnitude of tLTP. We found that the pairing protocol, which induced a strong tLTP in control slices, failed to elicit tLTP in slices pretreated with the MAGL inhibitor JZL184 (1 µM) (Control = 169.3 ± 7.7% of baseline, JZL184 = 113. 2 ± 8.8% of baseline, *n* = 9, *p* < 0.05, independent *t*-test, control vs. JZL184, Figure 4A). In contrast, inhibition of FAAH with PF750 (3 µM), did not alter the magnitude of the tLTP (Control = 169.3 ± 9.3% of baseline, PF750 = 162.2 ± 9.5% of baseline, *n* = 9, n. s, Control vs. PF750, Figure 4A). These results indicate that a transient increase in tonic 2-AG, but not AEA signaling, inhibits tLTP of glutamatergic synapses in the DRn.

A transient increase in tonic 2-AG levels activates CB1Rs, reduces glutamate release (Haj-Dahmane et al., 2018) and induces presynaptic LTD of glutamatergic synapses in the DRN (Haj-Dahmane and Shen, 2014). Consequently, it is possible that the blockade of tLTP by the increase in tonic 2-AG signaling could simply be attributed to a persistent depression of glutamate release, which masks tLTP induction. To test this possibility, we examined whether blockade of CB1Rs could rescue the tLTP in slices pretreated with JZL184. To that end, DRN slices were incubated (30–45 min) with JZL 184 in the presence of the CB1R antagonist/reverse agonist AM 251 (3 µM). Several hours (4–5 h) after washout, we assessed the magnitude of the tLTP. The results of this experiment showed that blocking CB1Rs did not rescue the tLTP (Control = 159.2 ± 8.9% of baseline; JZL184 + AM 251 = 99.8 ± 11.7% of baseline, *n* = 9, *p* < 0.02, independent *t*-test, control vs. JZL184 + AM 251, Figure 4B). We also examined the potential role of CB2Rs by testing whether blockade of CB2Rs with AM 630 (3 µM) could rescue the tLTP. We found that blocking CB2R failed to rescue the tLTP (JZL 184 = 109.2 ± 8.9% of baseline, *n* = 9; JZL 184 + AM 630 = 102.8 ± 7.7% of baseline, *n* = 9, n. s, data not shown). Collectively, these results indicate that the inhibition of the tLTP induced by a transient increase in tonic 2-AG signaling is not mediated by activation of CB1Rs or CB2Rs.

### Tonic 2-AG Signaling Controls tLTP *via* Peroxisome Proliferator-Activated Receptors γ and Nitric Oxide Signaling Pathways

**FIGURE 5:**
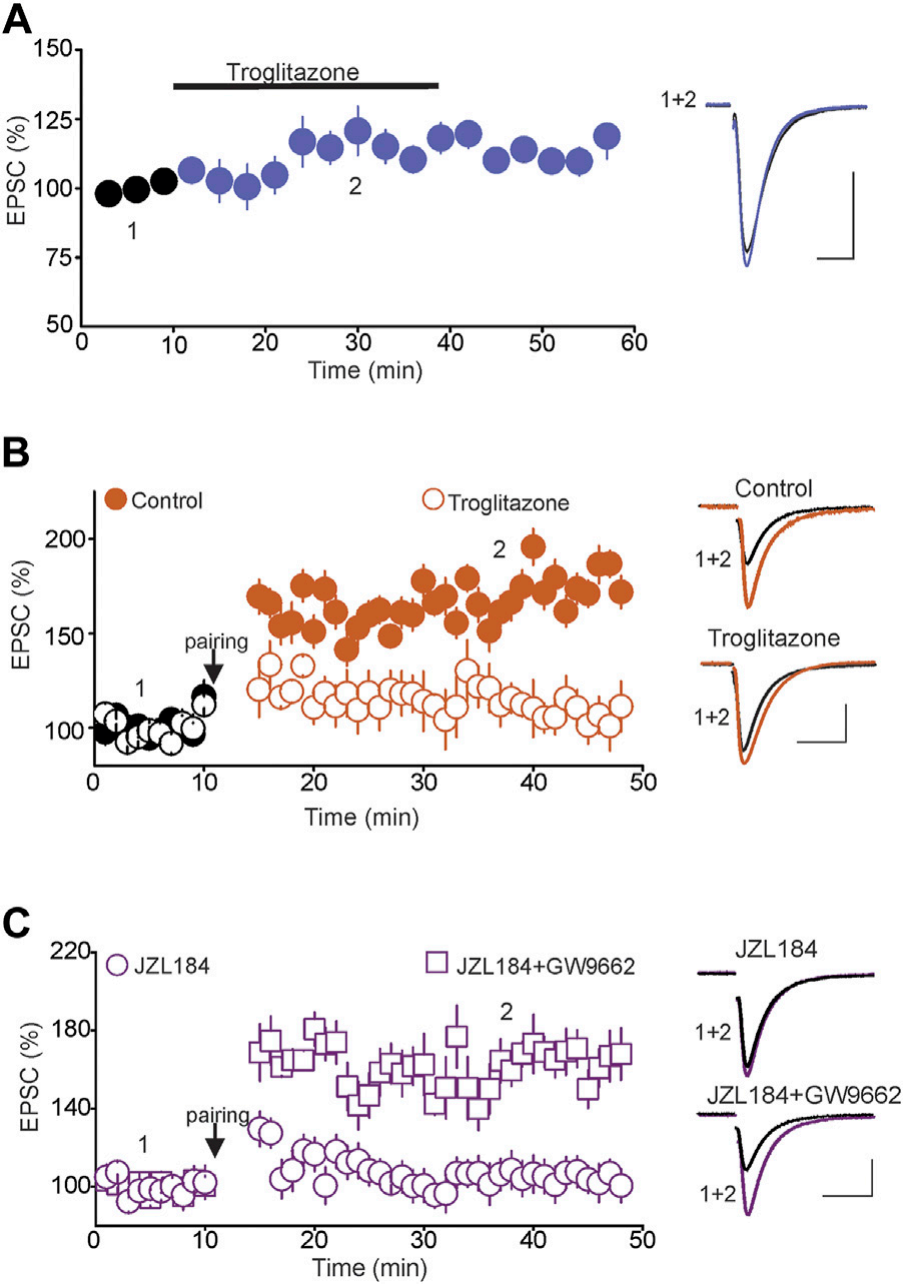
Tonic 2-AG signaling blocks tLTP *via* Activation of PPARγ. **(A)** Activation of PPARγ has no effect on the baseline amplitude of eEPSCs. Left panel illustrates the effect of troglitazone (10 µM; *n* = 9) on the amplitude of eEPSCs. Right graph is superimposed eEPSC traces taken before and during administration of troglitazone. Calibration bars: 100 pA, 10 ms. **(B)** Activation of PPARγ abolishes the tLTP. Left graph is a summary of tLTP obtained in control slices (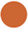; *n* = 11) and in slices treated with troglitazone (10 µM; 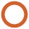; *n* = 11). Right graph illustrates sample eEPSC traces taken before and during the tLTP in control slices and slices treated with troglitazone. Calibration bars: 50 pA, 10 ms. **(C)** Blockade of PPARγ rescues the tLTP in slices incubated with JZL184. Left graph illustrates a summary of tLTP in slices incubated in JZL184 (1 µM; 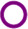; *n* = 10) and in JZL184 + GW9662 (10 µM; 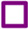; *n* = 10). Right graph illustrates superimposed eEPSCs traces at the time points indicated by numbers in the left panel. Calibration bars: 50 pA, 10 ms.

Results from previous studies have shown that 2-AG can also activate nuclear receptor protein peroxisome proliferator-activated receptors (PPARs), including PPARγ (Lenman and Fowler, 2007; Pertwee et al., 2010; Pistis and Melis, 2010), which are expressed in several brain areas, including the DRN (Sarruf et al., 2009; Schnegg and Robbins, 2011; Warden et al., 2016). Through this signaling pathway, 2-AG exerts both genomic (Du et al., 2011; Raman et al., 2011) as well as rapid non-genomic effects in the brain (Xu and Chen, 2015). Therefore, we hypothesized that activation of the PPARγ pathway could signal the blockade of tLTP induced by increased tonic 2-AG signaling. To test this hypothesis, we first examined whether activation of PPARγ using selective exogenous agonists could block the tLTP and mimic the effect of tonic 2-AG signaling. Remarkably, we found that although treatment of DRN slices with the PPARγ agonist troglitazone (10 µM) did not significantly alter the baseline amplitude of eEPSCs (105.2 ± 7.5% of baseline, *n* = 9, n. s. vs. baseline, Figure 5A), it abolished the tLTP (Control = 172.5 ± 8.5% of baseline, *n* = 11, *p* < 0.05 vs. baseline; Troglitazone = 107.5 ± 11.06% of baseline, *n* = 11, n. s. vs. baseline, Figure 5B) and mimicked the blockade of tLTP induced by the increase in tonic 2-AG signaling. Such results also indicate that the blockade of the tLTP in response to PPARγ activation could not be attributed to alterations of baseline glutamatergic synaptic transmission. Next, we tested whether blockade of PPARγ signaling could prevent the effect of the increased tonic 2-AG signaling and rescue the tLTP. To that end, we examined the magnitude of the tLTP in slices treated with JZL184, and JZL184 plus GW9662 (3 µM), a PPARγ antagonist. Consistent with an effect signaled by PPARγ, pretreatment of DRN slices with GW9662 prevented the blockade of tLTP induced by JZL184 (JZL184 = 104.5 ± 8.5% of baseline, *n* = 10, n. s. vs. baseline; JZL184 + GW9662 = 165.8 ± 11.06% of Baseline, *n* = 11, *p* < 0.05, paired *t*-test, vs. baseline, Figure 5C). Collectively, the findings that the PPARγ agonist and antagonist mimicked and prevented the blockade of the tLTP induced by JZL184, respectively, indicate that tonic 2-AG signaling inhibits tLTP induction *via* activation of PPARγ.

**FIGURE 6:**
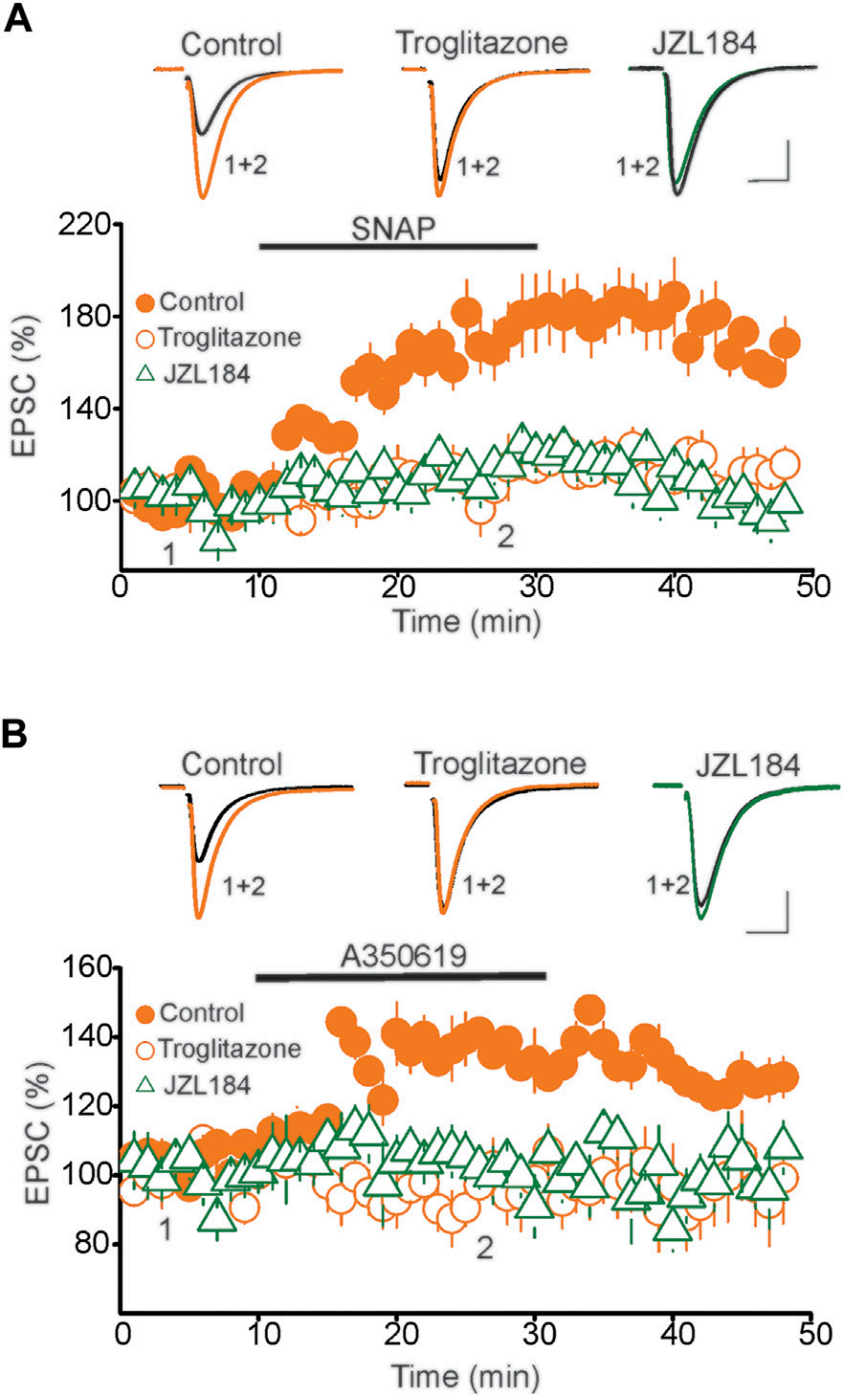
PPARγ abolishes the potentiation of eEPSCs induced by activation of NO/sGC pathway. **(A)** Pretreatment of DRn slices with PPARγ agonist troglitazone or MAGL inhibitor JZL184 blocks the SNAP-induced potentiation of eEPSCs. Lower graph illustrates a summary of the potentiation of eEPSCs induced by SNAP (200 µM) in control (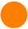; *n* = 9), and in slices pretreated with troglitazone (10 µM; 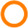; *n* = 10) or JZL 184 (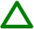; *n* = 10). Upper graph illustrates representative eEPSC traces collected before and during administration of SNAP. Calibration bars: 100 pA, 10 ms. **(B)** Troglitazone and JZL184 block the potentiation of eEPSCs induced by activation of soluble guanylate cyclase (sGC). Lower graph illustrates a summary of the potentiation of EPSCs induced by the sGC activator, A350619 (100 µM) obtained in control (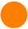; *n* = 9), and in slices pretreated with 10 µM troglitazone (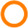; *n* = 10), or 1 µM JZL184 (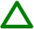). Upper panel represents superimposed eEPSC traces taken before and during administration of A350619 as indicated by numbers in the lower panel. Calibration bars: 25 pA, 10 ms.

A previous mechanistic study has shown that the tLTP of DRN glutamatergic synapses is mediated by a persistent increase in glutamate release (Haj-Dahmane et al., 2017). This form of presynaptic LTP is signaled by activation of the nitric oxide (NO)/cGMP/PKG signaling pathway (Haj-Dahmane et al., 2017). Interestingly, activation of PPARγ has been shown to regulate the NO/cGMP/PKG signaling cascade (Garcia-Bieno et al., 2005; Sadaghiani et al., 2011; Javadi et al., 2013), thereby raising the possibility that activation of PPARγ could mediate the blockade of tLTP by inhibiting the NO signaling pathway. To test this possibility, we conducted two sets of experiments. First, we examined the impact of an increase in tonic 2-AG levels using the MAGL inhibitor JZL184 on the potentiation of eEPSCs induced by the NO donor SNAP. Consistent with a previous report (Haj-Dahmane et al., 2017), in control slices, administration of SNAP (200 µM) potentiated the amplitude of EPSCs (177.7 ± 14.4% of baseline, *n* = 9, *p* < 0.01 paired *t*-test, vs. baseline, Figure 6A). Remarkably, SNAP-induced potentiation of eEPSCs was blocked in DRN slices pretreated with the MAGL inhibitor JZL184 (1 µM) (Control = 177.7 ± 14.4% of baseline, JZL184 = 117.2 ± 6.6% of baseline, *n* = 11, *p* < 0.02, independent *t*-test, JZL184 vs. Control, Figure 6A). Similarly, preincubation of DRN slices (30 min) with the PPARγ exogenous agonist troglitazone (10 µM) prevented the SNAP-induced potentiation of the eEPSC amplitude (Troglitazone = 112.5 ± 8.1% of baseline, *n* = 9, n. s. vs. baseline, Figure 6A). Collectively, these results suggest that activation of PPARγ either by exogenous agonists or by tonic 2-AG inhibits NO signaling, which in turn mediates the blockade of the tLTP.

Next, because NO potentiates the amplitude of eEPSCs through activation of soluble guanylate cyclase (sGC) and protein kinase G (PKG) pathways (Haj-Dahmane et al., 2017), we tested whether activation of PPARγ inhibits the sGC and PKG signaling cascade. To that end, the effects of a PPARγ agonist was examined on the potentiation of the eEPSC induced by activation of sGC/PKG pathway. Activation of sGC with A350619 (100 µM) potentiated the amplitude of eEPSCs in control (137.2 ± 5.4% of baseline, *n* = 9, *p* < 0.05, paired *t*-test, A350619 vs. baseline, Figure 6B), but not in DRN slices pretreated with troglitazone (94.8 ± 6.6% of baseline, *n* = 9, n. s. vs. baseline, Figure 6B). Activation of PPARγ with troglitazone also prevented the potentiation of eEPSCs induced by the PKG activator 8-pCPT-cGMP (8-pCPT-cGMP = 148.5 ± 7.5% of baseline, *p* < 0.05 vs. baseline; troglitazone + 8-pCPT-cGMP = 102.6 ± 6.5% of baseline, n. s. vs. baseline, *n* = 10, data not shown). Similarly, treatment of DRN slices with JZL184 (1 µM) abolished the potentiation of the eEPSC amplitude induced by the sGC activator A350619 (100 µM) (102.9 ± 6.6% of baseline, *n* = 9, n. s. vs. baseline, Figure 6B) and PKG activator 8-pCPT-cGMP (*n* = 10 neurons, data not shown). Collectively, these results suggest that activation of PPARγ blocks tLTP of DRN glutamatergic synapses by inhibiting the NO/cGMP/PKG signaling cascade.

## Discussion

While the detailed mechanisms and functional roles of phasic eCB signaling have been well characterized (Cachope, 2012; Castillo et al., 2012), very little is known about the functional roles of tonic eCB signaling and the mechanisms by which it controls synaptic function. Here, we show that glutamatergic synapses onto putative DRN 5-HT neurons are under the control of tonic 2-AG and AEA signaling. This tonic e-CB signaling, which controls baseline glutamatergic synaptic transmission *via* CB1Rs is tightly regulated by the basal neuronal network activity. Importantly, our results reveal that manipulations that induce short-term increases in tonic 2-AG, but not AEA signaling, impair Hebbian plasticity. This novel modulatory effect on synaptic plasticity is not signaled through canonical cannabinoid receptors, but by the activation of PPARγ and inhibition of the NO/cGMP/PKG signaling cascade. As such, this study unravels a novel function of tonic eCB signaling and expands the molecular mechanisms by which eCBs control synaptic transmission and plasticity in the brain.

### Baseline Network Activity Controls Tonic eCB Signaling in the DRN

The results of the present study show that blockade of CB1Rs readily increases glutamatergic synaptic transmission in the DRN, thereby demonstrating that tonic eCB signaling exerts an inhibitory effect on glutamatergic inputs onto putative DRN 5-HT neurons. Unlike GABA synapses of the hippocampal CA1 region, where tonic eCB signaling is attributed to constitutively active CB1Rs independent of endogenous agonists (Lee et al., 2015), here we show that the persistent CB1R activation is mediated by tonic eCB synthesis/release. This conclusion is supported by the observation that blockade of CB1Rs with the neutral CB1R antagonist NESS 0327 increases glutamatergic transmission to the same level as AM 251, an inverse CB1R agonist. In addition, pharmacological manipulations that inhibit 2-AG and AEA metabolism, which increase their synaptic levels, depress glutamatergic synaptic transmission onto DRN 5-HT neurons, through CB1R activation. The presence of tonic 2-AG and AEA mobilization is also in agreement with the earlier report of measurable levels of both AEA and 2-AG in unstimulated DRN brain slices (Haj-Dahmane et al., 2018) and with the ubiquitous role of tonic eCB mobilization in controlling basal synaptic transmission in other brain areas (Hentges et al., 2005; Neu et al., 2007; Roberto et al., 2010).

Examination of the mechanisms controlling tonic eCB signaling reveals that baseline neuronal activity tightly regulates tonic eCB synthesis/release. Thus, silencing neuronal activity by inhibiting action potential or blocking excitatory synaptic transmission, which has no significant effects on basal glutamate release and AMPAR function, profoundly reduces the magnitude of tonic eCB signaling. Interestingly, blockade of neuronal activity has also been shown to inhibit tonic AEA signaling at GABA synapses in hippocampal slice culture (Kim and Alger, 2010), further supporting the notion that constitutive eCB signaling is controlled by tonic neuronal activity. In the present study, we show the significant constitutive eCB signaling that persists following blockade of action potentials is abolished by blockade of glutamate receptors, indicating that eCB synthesis and release is mainly controlled by spontaneous and quantal excitatory synaptic transmission. Importantly, unlike phasic eCB synthesis/release, which is driven by a transient rise in postsynaptic [Ca ^2+^]_i_ (Wilson and Nicoll, 2001; Ohno-Shosaku and Kano, 2014), tonic eCB synthesis/release in the DRN does not require an increase in [Ca ^2+^]_i_. Indeed, manipulations that buffer postsynaptic [Ca ^2+^]_i_ did not block, but rather enhanced tonic eCB signaling. The unexpected increase in tonic eCB signaling induced by buffering postsynaptic [Ca ^2+^]_i_ could be attributed to alteration of eCB metabolism. Though, it is unknown whether the enzymatic activity of both FAAH and MAGL is regulated by [Ca ^2+^]_i_ **.** Collectively, these findings indicate that basal level of postsynaptic [Ca ^2+^]_i_ controlled by spontaneous excitatory drive is sufficient to induce a significant tonic eCB synthesis. Such a conclusion is consistent with the presence of a substantial amount of eCBs, including 2-AG in unstimulated brain, and after genetic and pharmacological inhibition of the diacylglycerol lipases (DGLs), the main enzymes of 2-AG synthesis (Gao et al., 2010; Tanimura et al., 2010; Haj-Dahmane et al., 2018). Importantly, the observations that tonic eCB mobilization involves different mechanisms than phasic eCB release suggests the presence of two distinct eCB pools that mediate tonic and phasic eCB signaling at central synapses (Alger and Kim, 2011). However, future studies are necessary to further support this notion.

In addition of controlling glutamatergic inputs onto DRN 5-HT neurons, we have previously shown that eCBs *via* activation of CB1Rs reduce the strength of glutamate synapses impinging onto DRN GABAergic neurons (Geddes et al., 2016). Furthermore, results from a previous study have suggested that CB1Rs control local GABAergic network (Mendiguren and Pineda, 2009). Consequently, it is tempting to speculate that tonic eCB signaling may also fine tune glutamate synapses impinging onto DRN GABAergic as well as GABA synapses onto 5-HT neurons. Clearly, additional studies are required to dissect the synapse and cell-specific effects of tonic eCB signaling in the DRN. The outcome of these studies will enhance the current understanding of eCB mediated modulation of local network activity in the DRN.

### PPARγ Signals the Effects of Tonic 2-AG on tLTP of DRN Glutamatergic Synapses

Beside controlling basal excitatory synaptic transmission, our results reveal that tonic 2-AG, but not AEA signaling, gates the plasticity of DRN 5-HT neurons glutamate synapses. Indeed, pharmacological manipulations that transiently increase tonic 2-AG levels prevent the tLTP. Interestingly, unlike phasic 2-AG-mediated long-term depression (LTD), which is signaling by CB1Rs (Haj-Dahmane and Shen, 2014; Haj-Dahmane et al., 2018), the blockade of the tLTP induced by tonic 2-AG signaling is not mediated by canonical CBRs (i.e. CB1Rs and CB2Rs), but rather by activation of PPARγ. This conclusion is supported by the findings that CB1R and CB2R antagonists fail to prevent the blockade of the tLTP. Whereas, blockade and activation of PPARγ abolishes and mimics the effect of tonic 2-AG signaling on the tLTP, respectively. The involvement of PPARγ in mediating the effects of tonic 2-AG on the tLTP is consistent with the growing evidence that some of the physiological effects of 2-AG, are mediated by activation of nuclear PPARγ (Pistis and Melis, 2010; Du et al., 2011; O’Sullivan, 2016). Activation of PPARγ has been shown to mediate, at least in part, the anti-inflammatory and neuroprotective effects of 2-AG (Du et al., 2011; Xu and Chen, 2015) by inhibiting the transcription of genes involved in neuroinflammatory processes (Du et al., 2011; Pistis and O’Sullivan, 2017). Consequently, these initial studies have led to the notion that eCB signaling through PPARγ is strictly involved in the regulation of neuroinflammation (Du et al., 2011; Xu and Chen, 2015; O’Sullivan, 2016). However, the present observation that tonic 2-AG gates synaptic plasticity *via* PPARγ expands the role of this signaling pathway to include the regulation of normal synaptic processing.

### Inhibition of NO/cGMP/PKG Signaling Mediates the Effect of PPARγ on the tLTP

Generally, activation of PPARγ controls the transcription of an array of genes, in particular genes that are involved in the regulation of lipid metabolism (Lehrke and Lazar, 2005), oxidative stress (Garcia-Bieno et al., 2005; Deng et al., 2020), and neuroinflammatory processes (Garcia-Bieno et al., 2005; Gray et al., 2012). In the present study, we show that PPARγ controls the tLTP of glutamatergic synapses by inhibiting the NO/cGMP/PKG signaling pathways. This conclusion is based on the observations that activation of PPARγ either by 2-AG or by exogenous PPARγ agonists prevents the potentiation of glutamatergic synapses induced by NO donors and sGC activators. Such findings indicate that the blockade of the tLTP by PPARγ is most likely mediated by an effect downstream from nitric oxide synthases (NOS), although alterations of the expression and the enzymatic activity of nNOS, iNOS or sGC cannot be excluded. Interestingly, activation of PPARγ has been shown to prevent the neurotoxic effects of NO donors on cortical neurons (Gray et al., 2012). Furthermore, results from previous studies have shown that some of the behavioral and electrophysiological effects of activation of PPARγ are mediated by inhibition of NO/cGMP/PKG signaling systems (Garcia-Bieno et al., 2005; Mohazab et al., 2012; Javadi et al., 2013). Collectively, these studies indicate that NO/cGMP/PKG signaling pathways play a prominent role in mediating the physiological effects of PPARγ.

At glutamatergic synapses of the DRN, both 2-AG and NO mediate retrograde modulation of synaptic plasticity. Thus, activity-driven, phasic 2-AG and NO release exert opposing effects on synaptic plasticity; with 2-AG inducing LTD (Haj-Dahmane and Shen, 2014) whereas NO mediating tLTP (Haj-Dahmane et al., 2017). The present finding that tonic 2-AG inhibits tLTP *via* PPARγ-dependent modulation of the NO/cGMP/PKG signaling cascade establishes a cross-talk between eCB and nitrergic systems in controlling synaptic plasticity in the DRN. Because the magnitude of tonic 2-AG signaling is tightly regulated by basal neuronal activity, it is possible that the enhanced tonic 2-AG signaling during neuronal activation could function as a feedback control that prevents excessive network activity by blocking tLTP *via* PPARγ-mediated inhibition of NO/cGMP/PKG signaling. Conversely, chronic network inactivity, which leads to a reduced tonic 2-AG signaling and decreased PPARγ activation will facilitate NO-mediated potentiation of glutamate synapses. Accordingly, in this model, tonic 2-AG signaling through PPARγ and NO pathways may play a key role in controlling synaptic homeostasis and scaling in the DRN and other brain areas. As such, alterations of tonic 2-AG signaling reported in neurological/psychological disorders, such as autism, may lead to abnormal synaptic homeostasis and scaling. It is noteworthy that previous studies using genetic and environmental animal models of autism have reported a reduced constitutive eCB tone (Földy et al., 2013; Kerr et al., 2013; Chakrabarti et al., 2015; Brigida et al., 2017), which has been shown to mediate, at least in part, the abnormal synaptic homeostasis (Speed et al., 2015; Kuo and Liu, 2018; Wang et al., 2018) and the associated behavioral deficits (Chakrabarti et al., 2015; Kuo and Liu, 2018). However, future studies are required to test whether these synaptic and behavioral effects are mediated by altered PPARγ and NO signaling pathways.

### Significance Statement

Endocannabinoids (eCBs) modulate a plethora of physiological and behavioral processes *via* phasic and tonic signaling at central synapses. Altered eCB signaling is associated with numerous neurological disorders, such as autism and epilepsy. While the functions of phasic eCB signaling are well characterized, the physiological roles of tonic eCB signaling remain not well defined. Here, we show that in the dorsal raphe nucleus (DRN), tonic eCB signaling gates synaptic plasticity. This regulatory effect is not mediated by classical cannabinoid receptors (CBRs), but by a novel mechanism that involves intracellular peroxisome proliferator-activated receptor gamma (PPARγ) and inhibition of nitric oxide (NO) signaling cascade.

## Data Availability

The raw data supporting the conclusions of this article will be made available by the authors, without undue reservation.
